# Application for Neuralgia

**Published:** 1886-01-20

**Authors:** 


**Application for Neuralgia.**—An excellent local application for the relief of neuralgia and gout is prepared by rubbing up together equal parts of thymol, menthol, camphor and chloral.—*N. E. Med. Monthly.*

